# SARS-CoV-2 Neutralizing Antibodies 2.0

**DOI:** 10.3390/v16111791

**Published:** 2024-11-19

**Authors:** Youchun Wang

**Affiliations:** Institute of Medical Biology, Chinese Academy of Medical Sciences & Peking Union Medical College, Kunming 650118, China; wangyc@imbcams.com.cn

As the SARS-CoV-2 mutates, especially into those variants causing immune escape, COVID-19 continues to wreak havoc. The world is in dire need of figuring out the correlation between neutralizing antibodies and protection mechanisms, and of developing effective therapeutic and prophylactic products against emerging variants. In this Special Issue 2.0, we present to the readers of *Viruses* one brief report, two reviews, one communication, and seven articles focusing on the study of promising antibody and vaccine candidates and desirable detection assays and research platforms. 

Vaccines and antibody treatments have mitigated the spread of COVID-19 over the past 4 years. We are delighted to see an array of antibodies and vaccines remaining effective in dealing with the latest variants. Through categorization, Cui et al. provided a comprehensive review of broadly neutralizing antibodies that retain effectiveness against the currently dominating variants [1]. Chen et al. reported an optimized neutralizing antibody Ab08-K99E that bears the L452R mutation [2]. The study from Miyashita et al. demonstrated the clinical efficacy of Sotrovimab in addressing Omicron BA.1 and BA.2 infections [3]. Godínez-Palma et al. revealed that IgG-A7 at an appropriate dose still possesses a broad efficacy profile [4]. The llama-derived nanobodies developed by Pavan et al. might also be the potential candidates for the COVID-19 treatment, especially with the cocktail therapy [5]. Except for the antibody treatment, Widyasari et al. found that bivalent mRNA booster vaccination could protect against the circulating Omicron variants regardless of infection history [6].

As time goes by, the once promising antibody MTX-COVAB prepared by Hillenbrand et al. has lost its efficacy. However, their experience in constructing the DROPZYLLA® platform sheds light on the rapid identification of antibodies [7]. The works from Rocha et al. and Thimmiraju et al. are highly instructive for practical use, in which the former reviewed different methods for measuring the neutralizing antibody titers [8] and the latter introduced parameters that can improve the transduction efficiency of the HIV-based pseudovirus platform [9].

When it comes to real-world use, Vargas-De-León et al. found that infection history and comorbidities have a certain influence on antibody generation and the response to the vaccine [10]. The study conducted by Radion et al. suggested that after vaccination with Sputnik V, discrepancies exist regarding the temporal dynamics of neutralizing and non-neutralizing antibodies [11]. 

During preparation, we are pleased to note that several studies have integrated cutting-edge technologies into research. The wide use of pseudotyped viruses as the surrogate for authentic viruses provides unparalleled convenience, serving as an important tool for studying SARS-CoV-2 [12]. It is the revolutionized technologies represented by the structure-based design and computational approach that have guaranteed the rapid and successful development of COVID-19 vaccines and therapies [13,14]. Undoubtedly, all the above aspects will be the backbone for future development and beneficial to the rapid pandemic response. 

Furthermore, recognizing the decreased cellular immunity and the Fc-mediated roles of antibodies is fundamental to comprehending the sustained immune protection against COVID-19 [13]. In this way, it is noteworthy that some researchers have touched on another protection mechanism, i.e., Fc-effector functions, which may act in synergy with neutralizing antibodies and pave the way for more effective treatment. 

It is undeniable that current studies have certain limitations in terms of study design, sample size, etc. In addition, the efficacy and effectiveness of both antibody therapies and vaccines need to be validated in the clinical study and real world for a longer period of time together with the ever-changing setting. 

In closing, we would like to thank all the researchers for their unremitting efforts in yielding these encouraging results. Further research and development concerning SARS-CoV-2 neutralizing antibodies and relevant assays are highly welcomed in the post-pandemic era to ensure we are fully prepared for the next pandemic. 
